# Knockout of *Trpa1* accelerates age-related cardiac fibrosis and dysfunction

**DOI:** 10.1371/journal.pone.0274618

**Published:** 2022-09-14

**Authors:** Shuangtao Ma, Donna H. Wang

**Affiliations:** Division of Nanomedicine and Molecular Intervention, Department of Medicine, College of Human Medicine, Michigan State University, East Lansing, Michigan, United States of America; INSERM, Université de Bordeaux, FRANCE

## Abstract

Age-related cardiac fibrosis contributes to the development of heart failure with preserved ejection fraction which lacks ideal treatment. Transient receptor potential ankyrin 1 (TRPA1) is an oxidative stress sensor and could attenuate age-related pathologies in invertebrates. The present study aimed to test whether TRPA1 plays a role in age-related cardiac remodeling and dysfunction. The cardiac function and pathology of 12-week-old (young) and 52-week-old (older) *Trpa1*^-/-^ mice and wild-type (WT) littermates were evaluated by echocardiography and histologic analyses. The expression levels of 84 fibrosis-related genes in the heart were measured by quantitative polymerase chain reaction array. Young *Trpa1*^-/-^ and WT mice had similar left ventricular wall thickness, volume, and systolic and diastolic function. Older *Trpa1*^-/-^ mice had significantly increased left ventricular internal diameter and volume and impaired systolic (lower left ventricular ejection fraction) and diastolic (higher E/A ratio and isovolumetric relaxation time) functions compared with older WT mice (*P*<0.05 or *P*<0.01). Importantly, older *Trpa1*^-/-^ mice had enhanced cardiac fibrosis than older WT mice (*P*<0.05) while the two strains had similar degree of cardiac hypertrophy. Among the 84 fibrosis-related genes, *Acta2*, *Inhbe*, *Ifng*, and *Ccl11* were significantly upregulated, while *Timp3*, *Stat6*, and *Ilk* were significantly downregulated in the heart of older *Trpa1*^-/-^ mice compared with older WT mice. Taken together, we found that knocking out *Trpa1* accelerated age-related myocardial fibrosis, ventricular dilation, and cardiac dysfunction. These findings suggest that TRPA1 may become a therapeutic target for preventing and/or treating cardiac fibrosis and heart failure with preserved ejection fraction in the elderly.

## Introduction

Heart failure is a leading cause of death worldwide and affects more than 10% of people over 80 years of age [1]. Age is an independent risk factor for heart failure, especially for heart failure with preserved ejection fraction (HFpEF). Patients with HFpEF have clinical manifestations of heart failure due to increased left ventricular filling pressure with normal left ventricular ejection fraction (≥50%). HFpEF is primarily a disease of the elderly, and the majority of patients with HFpEF age 65 years and older [2]. Oxidative stress is gradually enhanced in the aging heart [3]. Excessive reactive oxygen species (ROS) production in oxidative stress promotes age-related reactive fibrosis in the heart through modulating both collagen synthesis and degradation [4]. Reactive fibrosis in the aging heart reduces ventricular compliance and impairs ventricular filling during diastole, leading to the development of diastolic dysfunction and HFpEF in the elderly [5]. Unlike heart failure with reduced ejection fraction, HFpEF, which represents approximately 50% of heart failure cases, currently lacks effective therapy [1].

Although oxidative stress plays a critical role in the development of cardiac fibrosis and HFpEF, antioxidants failed to improve the outcome of patients with HFpEF [6]. This calls for further studies on the regulation of ROS-induced cardiac fibrosis in aging. Transient receptor potential ankyrin 1 (TRPA1) is a calcium-permeable cation channel receptor responsible for detecting ROS overproduced in oxidative stress [7]. TRPA1 abundantly and functionally expresses in the plasma membrane of fibroblasts and may be activated by ROS [8, 9]. After activated by ROS, TRPA1 may inhibit ROS-induced apoptosis and ameliorate aging-related pathologies in invertebrates, such as *C*. *elegans* [10–13]. However, the role of TRPA1 in age-related disorders in mammals is largely unknown. A previous study demonstrates that TRPA1 receptors play a protective role in age-related endothelial dysfunction in mice by suppressing oxidative stress [13], suggesting an anti-aging effect of TRPA1 in the cardiovascular system.

In the present study, we aimed to investigate the role of TRPA1 in age-related cardiac remodeling and function in mice. We examined and compared the cardiac function and pathology of young and aged *Trpa1* gene knockout (*Trpa1*^-/-^) mice and their wild-type (WT) littermates. In addition, the expression profiling of profibrotic and antifibrotic genes was measured to identify genetic signature involved in the fibrosis-regulatory effects of TRPA1.

## Methods

### Animals

Male and female *Trpa1* heterozygous (*Trpa1*^+/-^) mice on the C57BL/6 genetic background were purchased from The Jackson Laboratory (Bar Harbor, ME, USA) and were crossbred to obtain homozygous *Trpa1* gene knockout (*Trpa1*^-/-^) mice and their WT (*Trpa1*^+/+^) control littermates [14]. Genotyping was performed by polymerase chain reaction (PCR) using genomic DNA extracted from the mouse-tail tissue with the primers listed on the website of The Jackson Laboratory. Mice were housed under a 12h light/12h dark cycle with free access to normal chow diet and drinking water. Twelve-week-old (young) and 52-week-old (older, equivalent to 42.5 human years) male and female mice were used in the experiments. This study was approved by the Institutional Animal Care and Use Committee of Michigan State University.

### Mouse echocardiography

Echocardiography was performed using an ultrasound imaging system (Vevo 2100; VisualSonics, Toronto, Canada). Measurement and data analysis were performed blinded. Anesthesia was induced in a chamber with 4–5% isoflurane and maintained at 1.5–2%. Mice were positioned supine on a heated platform to keep body temperature at 38°C. Ultrasound gel was applied to the prepared chest skin, then transthoracic echocardiogram was performed to assess cardiac structure and function. Two-dimensional images of the parasternal long-axis view and M-mode images of the short-axis view at the papillary muscle level were recorded. Transmitral flow was also recorded in the apical four-chamber view with a Doppler probe. We measured structural parameters, including left ventricular posterior wall thickness at end-diastole (LVPWd) and end-systole (LVPWs), left ventricular anterior wall thickness at end-diastole (LVAWd) and end-systole (LVAWs), left ventricular internal diameter at end-diastole (LVIDd) and end-systole (LVIDs), and left ventricular end-diastolic (LV Vol,d) and end-systolic (LV Vol,s) volume. We also calculated functional parameters, including left ventricular ejection fraction (LVEF), left ventricular fractional shortening (LVFS), the ratio of early to late diastolic filling (E/A), and isovolumetric relaxation time (IVRT).

### Pathology

At the end of the experiments, mice were euthanized by deep anesthesia with isoflurane (5%) to alleviate suffering, and then the hearts were harvested. The mouse hearts at the papillary muscle level were fixed in 4% paraformaldehyde, dehydrated, embedded in paraffin, and sectioned into 5μm-thick slides. Sections were stained with hematoxylin and eosin, and images were captured using a microscopy (Nikon TE2000-U, Melville, NY, USA). The cardiomyocyte size was determined by measuring the cross-sectional area using the ImageJ software (NIH). We performed Picro-Sirius Red stain to quantify cardiac fibrosis and collagen deposition.

### Quantitative polymerase chain reaction (PCR) arrays

The gene expression levels of 84 fibrosis-related genes were measured by quantitative PCR array assays in 96-well plates using RT^2^ Profiler PCR Array (Mouse Fibrosis, Cat. no. 330231 PAMM-120ZR, Qiagen, Valencia, CA, USA) according to the manufacturer’s protocol. The mRNA expression levels were calculated by the 2^−ΔΔCT^ method.

### Western blotting

Total proteins were extracted from the myocardial tissue and separated by electrophoresis using a 10% sodium dodecyl sulfate polyacrylamide gel. The gel was then transferred to a polyvinylidene difluoride membrane (SLGP033RS, Millipore, Bedford, MA, USA). The membrane was blocked with 5% bovine serum albumin for 1h and then incubated with anti-TRPA1 (1:1000 dilution, NB110-40763, Novus Biologicals, Littleton, CO, USA) or anti-GAPDH (1:1000 dilution, GTX627408-01, GeneTex, Irvine, CA, USA) antibodies overnight. The membrane was rinsed and then incubated with horseradish peroxidase-conjugated secondary antibodies for 1h at room temperature. The protein bands in the blot were detected using an enhanced chemiluminescence kit (Bio-Rad, Hercules, CA, USA) according to the manufacturer’s instructions. Relative density was measured using ImageJ software (NIH).

### Statistical analysis

Data are presented as means ± SE and analyzed using GraphPad prism software (San Diego, CA, USA). Comparisons among multiple groups were performed using two-way ANOVA with post hoc Tukey honestly significant difference test. Comparisons between two groups were performed with Student’s t-test. *P* values lower than 0.05 were considered statistically significant.

## Results

### TRPA1 is upregulated in the heart of older mice

**Fig 1:**
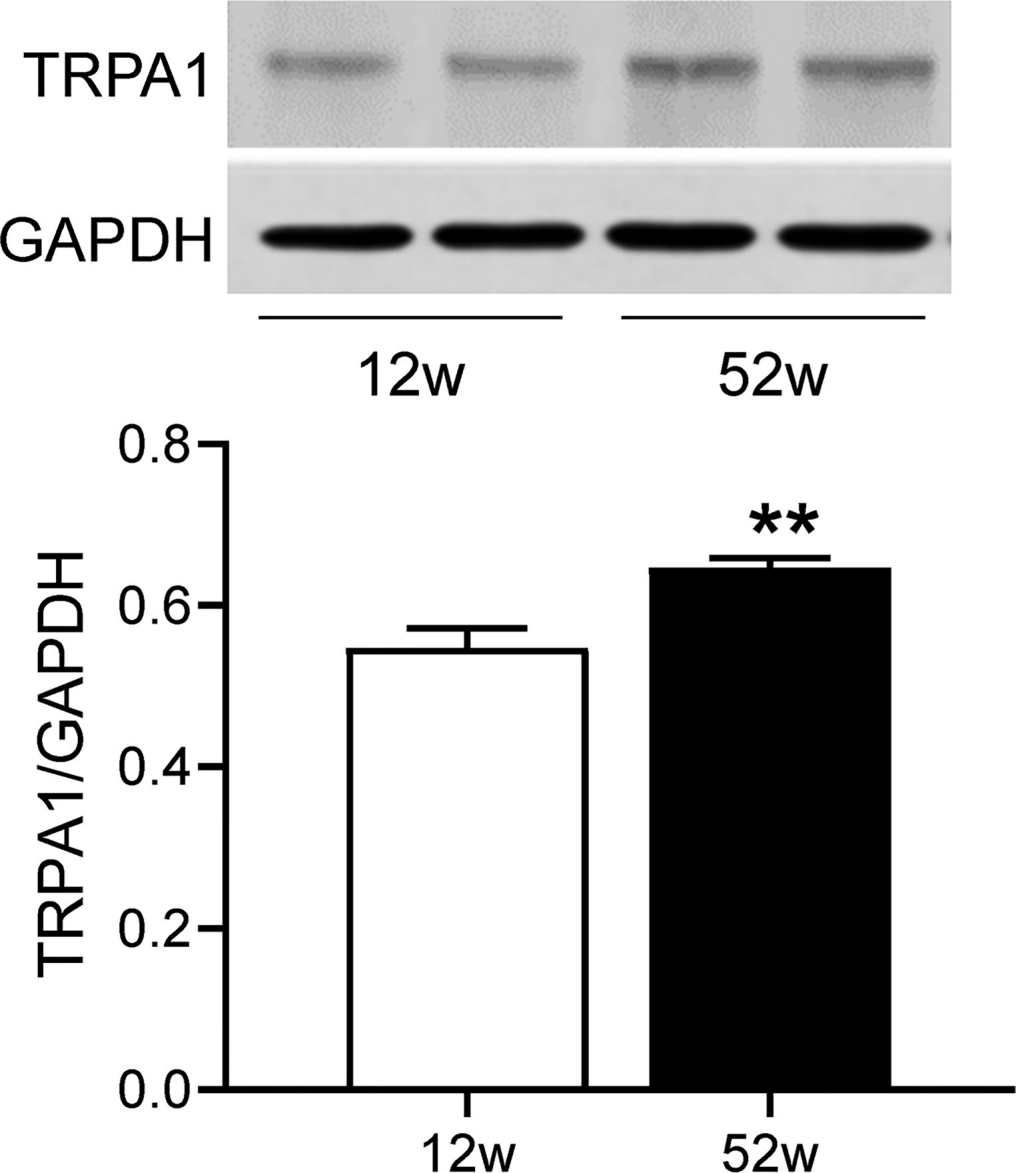
The TRPA1 protein level in the heart from older mice is decreased. The protein levels of TRPA1 were measured by Western blotting in the myocardial tissue from 12-week-old (young) and 52-week-old (older) wild-type mice. Data are mean ± SE. n = 4 per group. **P<0.01 *vs*. 12-week-old mice.

The protein expression of TRPA1 in the heart of 52-week-old (older) WT mice was significantly increased compared to that in the heart of 12-week-old (young) mice (*P*<0.01, Fig 1).

### Knockout of *Trpa1* enhances age-related left ventricular dilation and dysfunction

**Fig 2:**
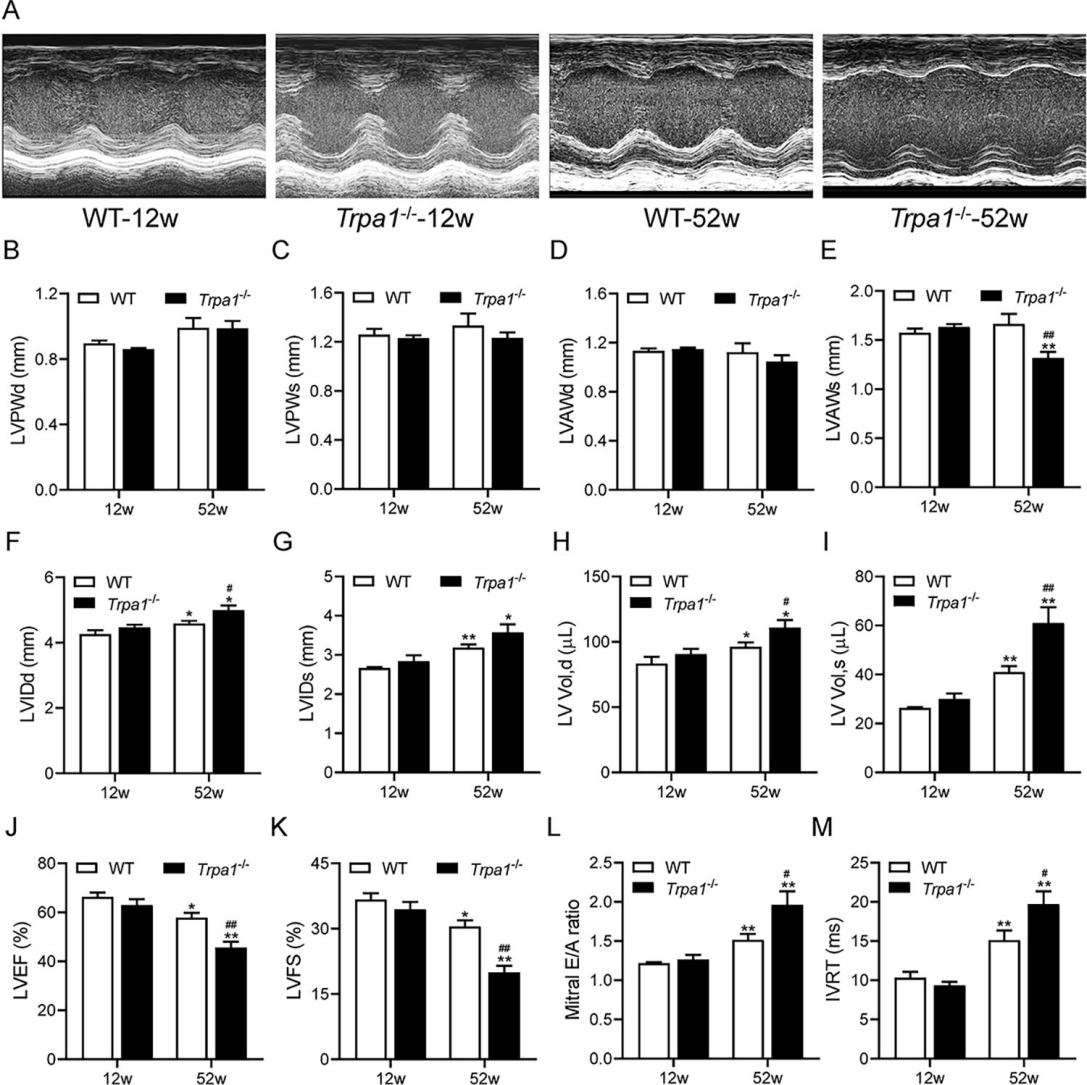
Knockout of *Trpa1* enhances age-related left ventricular dilation and dysfunction. (A) Representative M-mode echocardiograms of 12-week-old (young) and 52-week-old (older) *Trpa1*^-/-^ mice and WT littermates. The left ventricular posterior wall thickness at end-diastole (LVPWd) (B), left ventricular posterior wall thickness at end-systole (LVPWs) (C), left ventricular anterior wall thickness at end-diastole (LVAWd) (D), left ventricular anterior wall thickness at end-systole (LVAWs) (E), left ventricular internal dimension at end-diastole (LVIDd) (F), left ventricular internal dimension at end-systole (LVIDs) (G), left ventricular volume at end-diastole (LV Vol, d) (H), left ventricular volume at end-systole (LV Vol, s) (I), left ventricular ejection fraction (LVEF) (J), left ventricular fractional shortening (LVFS) (K), mitral E/A ratio (L), and isovolumetric relaxation time (IVRT) (M) of 12-week-old and 52-week-old *Trpa1*^-/-^ mice and WT littermates were measured by echocardiography. Data are mean±SE of 10 mice in each older mouse group and 6 mice in each young mouse group. *P<0.05, **P<0.01 *vs*. 12-week-old congenic mice; ^#^*P*<0.05, ^##^*P*<0.01 *vs*. 52-week-old WT mice.

Echocardiography was performed in 12-week-old (young) and 52-week-old (older) *Trpa1*^-/-^ and WT mice. Young *Trpa1*^-/-^ and WT mice had similar left ventricular wall thickness, internal diameter, and volume (Fig 2A–2I). Older *Trpa1*^-/-^ and WT mice had similar left ventricular wall thickness (Fig 2A–2I) except that older *Trpa1*^-/-^ mice had thinner LVAWs than older WT mice (*P*<0.05, Fig 2E). Both older *Trpa1*^-/-^ and WT mice had significantly larger left ventricular internal diameter and volume than their young congenic counterparts (*P*<0.05 or *P*<0.01, Fig 2A–2I). In addition, older *Trpa1*^-/-^ mice had significantly increased left ventricular internal diameter and volume when compared with older WT mice (*P*<0.05 or *P*<0.01, Fig 2F, 2H and 2I). Left ventricular systolic and diastolic functions were similar between young *Trpa1*^-/-^ and WT mice and were significantly impaired in their older congenic counterparts with lower LVEF and LVFS and higher E/A ratio and LVRT (*P*<0.05 or *P*<0.01, Fig 2J–2M). Moreover, older *Trpa1*^-/-^ mice had significantly decreased systolic (lower LVEF and LVFS) and diastolic (higher E/A ratio and LVRT) functions than older WT mice (*P*<0.05 or *P*<0.01, Fig 2J–2M).

### Knockout of *Trpa1* exacerbates age-related cardiac fibrosis

**Fig 3:**
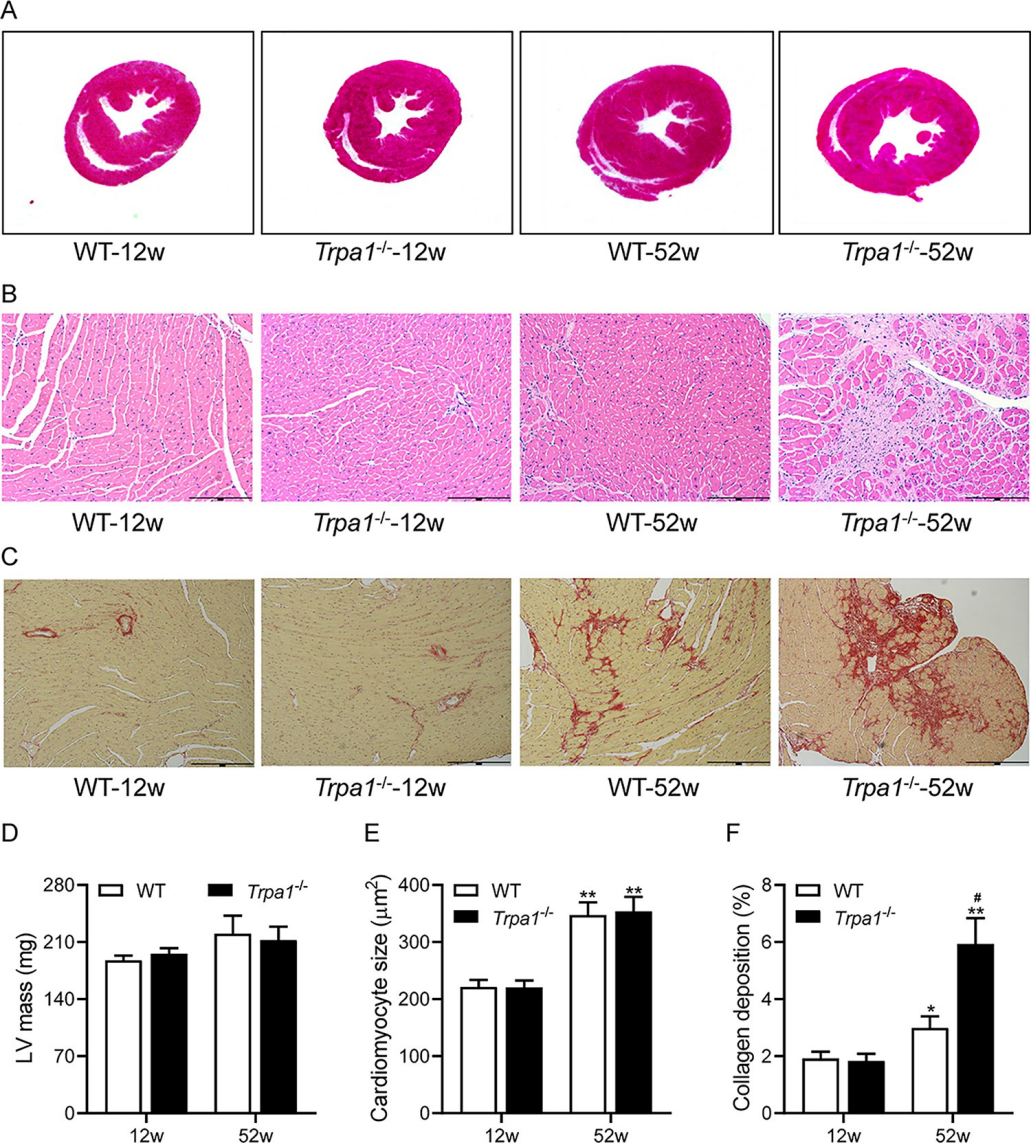
Knockout of *Trpa1* exacerbates age-related cardiac fibrosis. Representative H&E-stained whole-heart cross-sections (A) and H&E-stained (B) and Picrosirius red-stained (C) left ventricular sections of 12-week-old and 52-week-old *Trpa1*^-/-^ mice and WT littermates. (D) Left ventricular mass was evaluated by echocardiography. Cross-sectional area of cardiomyocytes (E) and collagen deposition fraction (F) were quantified based on H&E and PSR staining, respectively. Data are mean±SE of 6 mice in each group. *P<0.05, **P<0.01 *vs*. 12-week-old congenic mice; ^#^*P*<0.05 *vs*. 52-week-old WT mice.

Representative heart sections show that older *Trpa1*^-/-^ and WT mice had slightly hypertrophied hearts when compared with their young congenic counterparts and that older *Trpa1*^-/-^ mice had a dilated left ventricle when compared to older WT mice (Fig 3A). However, left ventricular mass estimated by echocardiography was similar between young *Trpa1*^-/-^ and WT mice and between older *Trpa1*^-/-^ and WT mice (Fig 3D). The size of cardiomyocytes was larger in older *Trpa1*^-/-^ and WT mice than their young congenic counterparts (both *P*<0.01), while the cardiomyocyte size was similar between older *Trpa1*^-/-^ and WT mice (Fig 3B and 3E). Older *Trpa1*^-/-^ and WT mice had significant cardiac fibrosis when compared with their young congenic counterparts (*P*<0.05 or *P*<0.01), and older *Trpa1*^-/-^ mice had enhanced cardiac fibrosis than older WT mice (*P*<0.05, Fig 3C and 3F).

### Knockout of *Trpa1* alters expression of fibrosis-related genes

**Fig 4:**
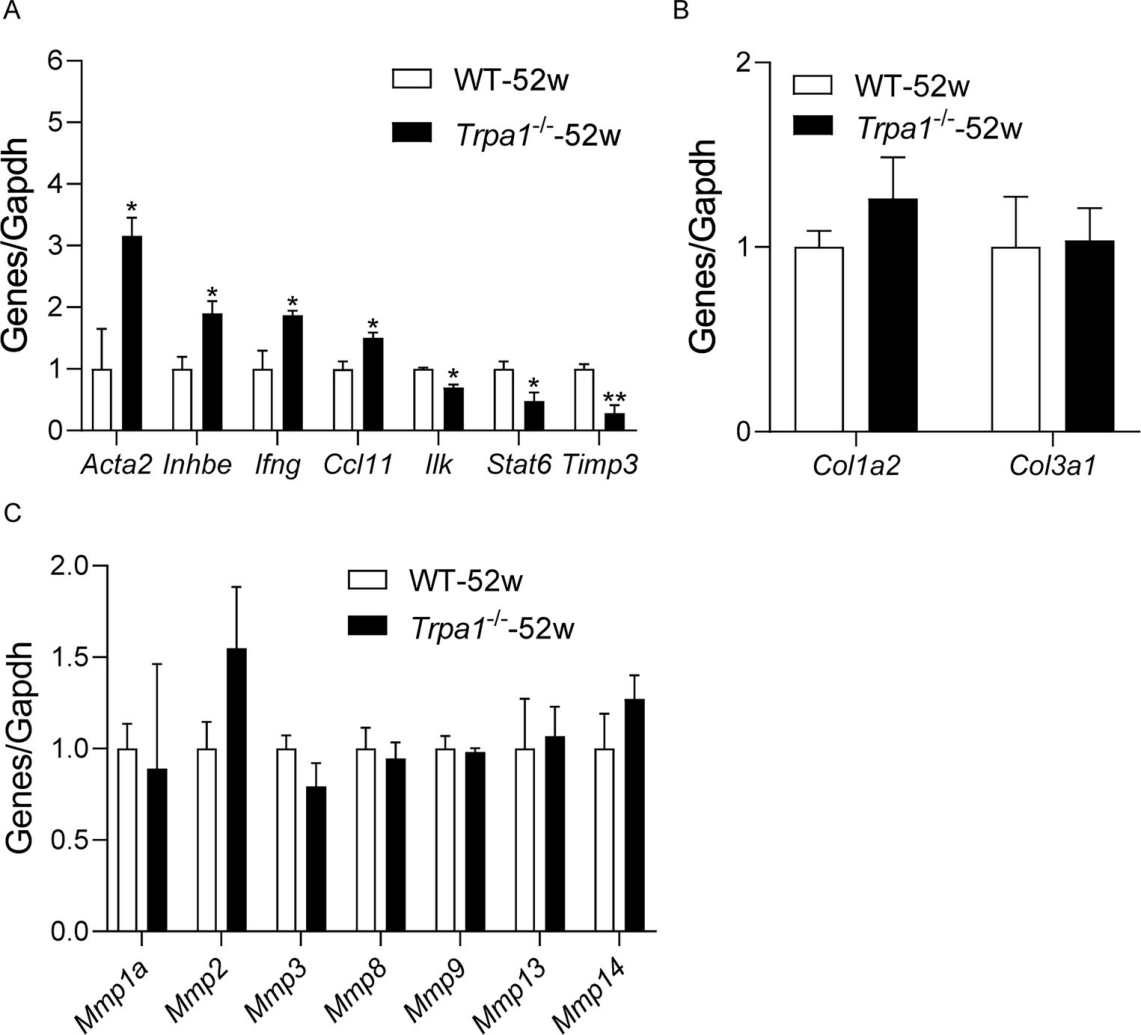
Knockout of *Trpa1* alters expression of fibrosis-related genes. Differently expressed genes (A), gene expression of collagens (B), and gene expression of matrix metalloproteinases (C) in the heart tissue 12-week-old and 52-week-old *Trpa1*^-/-^ mice and WT littermates were measured by quantitative PCR array assays. Data are mean±SE of 3 samples in each group. *P < 0.05, **P < 0.01 *vs*. 52-week-old WT mice.

We performed quantitative PCR array analyses of the mRNA expression of 84 fibrosis-related genes in the myocardial tissue of 52-week-old older *Trpa1*^-/-^ and WT mice. Among the 84 genes, only seven genes had significantly different expression levels in the heart between older WT and *Trpa1*^-/-^ mice (Fig 4A). The expression levels of *Acta2*, *Inhbe*, *Ifng*, and *Ccl11* were significantly increased with fold changes of 3.1, 1.9, 1.9, and 1.5 (all *P* < 0.05), respectively, while *Timp3*, *Stat6*, and *Ilk* were significantly decreased with fold changes of 0.3, 0.5, and 0.7 (*P* < 0.05 or *P* < 0.01), respectively, in the heart of older *Trpa1*^-/-^ mice compared with WT mice. The mRNA levels of *Col1a2* and *Col3a1*, markers of collagen synthesis, in the myocardial tissue were similar between older *Trpa1*^-/-^ and WT mice (Fig 4B). Matrix metalloproteinases (MMPs) are major enzymes responsible for degradation of collagen fibers. The mRNA levels of matrix metalloproteinases, including *Mmp1a*, *Mmp2*, *Mmp3*, *Mmp8*, *Mmp9*, *Mmp13*, and *Mmp14*, in the heart were similar between older *Trpa1*^-/-^ and WT mice (Fig 4C).

## Discussion

The present study aimed to determine the role of the TRPA1 channel in age-related changes of cardiac structure and function. We found that knockout of *Trpa1* exacerbated age-related myocardial fibrosis, ventricular dilation, and cardiac dysfunction in 52-week-old older mice. We also identified several genes which were involved in the exacerbated cardiac fibrosis due to TRPA1 ablation. These findings suggest that TRPA1 may act as a brake on age-related cardiac fibrosis, dilation, and dysfunction.

There are two types of cardiac fibrosis: reparative and reactive fibrosis. Reparative fibrosis is responsible for cardiac wound healing and repair after myocardial infarction, while reactive fibrosis refers to interstitial fibrosis in response to aging or hypertension. Aging impairs reparative fibrosis but enhances reactive fibrosis [4], and the enhanced reactive cardiac fibrosis in aging contributes to the development of HFpEF in the elderly [4, 5]. Reparative fibrosis is mainly due to activation of fibroblasts and increased synthesis of collagens, while age-related reactive fibrosis is majorly caused by decreased degradation of collagens [4, 15]. Age-related fibrosis is a reactive fibrotic response to oxidative stress [4]. ROS overproduced in oxidative stress promotes collagen deposition in the aging heart [16]. TRPA1 as an oxidative stress sensor has been shown to play important roles in pathophysiology of the cardiovascular system [17]. However, its role in cardiovascular aging is largely unknown. Our pilot study found that TRPA1 was upregulated in middle-aged mice at 52 weeks of age but was dramatically decreased in aged mice at 104 weeks of age (data not shown). This finding suggests that the upregulation of TRPA1 at middle age might be a compensation against age-related cardiac fibrosis. The present study using a *Trpa1*^-/-^ mouse model provides evidence that TRPA1 may play a protective role in age-related myocardial fibrosis, ventricular dilation, and cardiac dysfunction.

TRPA1 was reported previously to be involved in reparative fibrosis after myocardial infarction and pressure overload-induced cardiac hypertrophy likely through regulating fibroblast activation [18]. Their results suggest that TRPA1 could activate fibroblasts and might contribute to myocardial repair after infarction. Another study demonstrated that inhibition of TRPA1 suppressed pressure overload-induced cardiac fibrosis in a mouse model by negatively regulating Ca^2+^-dependent signal pathways [19]. Our results in the present study appear to conflict with previous results. The possible reason for the conflict may be that the mechanisms leading to cardiac fibrosis in cardiac repair after myocardial infarction, hypertension, and aging are different. In contrast to cardiac repair after myocardial infarction and hypertensive heart disease where fibroblast activity and collagen expression are markedly elevated, increased collagen synthesis is not the main culprit of fibrosis in the aging myocardium [4]. In fact, fibroblast activity and collagen synthesis are significantly impaired in the aging hearts, rather, collagen buildup and subsequent cardiac fibrosis in the aging heart is due to attenuation of matrix-degrading pathways [4]. Therefore, TRPA1 likely plays a protective role in age-related cardiac fibrosis through inhibiting collagen deposition while may contribute to cardiac fibrosis after myocardial infarction and in the setting of hypertension through activating fibroblasts.

The underlying mechanism for the regulatory role of TRPA1 cardiac fibrosis remains elusive. Hypertension is a common cause for cardiac fibrosis. Previous studies from our group and others demonstrated that TRPA1 plays little role in blood pressure regulation [20–22]. Therefore, the enhanced cardiac fibrosis in older *Trpa1*^-/-^ mice is unlikely due to changes in blood pressure. In order to reveal the potential mechanism and genetic signature of the accelerated cardiac fibrosis in older *Trpa1*^-/-^ mice, we performed quantitative PCR array analyses of the mRNA expression of 84 fibrosis-related genes in the myocardial tissue. The mRNA levels of *Col1a2* and *Col3a1* in the myocardial tissue were similar between older *Trpa1*^-/-^ and WT mice, suggesting comparable cardiac collagen synthesis in the two strains. In addition, the mRNA levels of matrix metalloproteinases, enzymes responsible for degradation of collagen fibers, in the heart were similar between older *Trpa1*^-/-^ and WT mice. These results suggest that the accelerated cardiac fibrosis in older *Trpa1*^-/-^ is due to neither increased collagen synthesis nor decreased collagen degradation. Among seven differentially expressed genes, *Timp3* is most significantly decreased in the heart of older *Trpa1*^-/-^ mice compared with WT mice. TIMP3 is an extracellular protein mainly secreted by fibroblasts and is involved in the post-translational modification and stabilization of collagen fibers [23]. Previous studies demonstrate that loss of *Timp3* accelerates reactive interstitial cardiac fibrosis likely through lysyl hydroxylase 1-mediated hydroxylation and stabilization of collagens [24, 25]. Loss of *Timp3* also promotes age-related renal fibrosis [26]. Therefore, downregulation of TIMP3 might stabilize extracellular collagen fibers and contribute to the accelerated cardiac fibrosis in older *Trpa1*^-/-^ mice. However, how TRPA1 regulates the expression of TIMP3 remains unclear. One of the limitations of the present study is that the protein expression and activity of TIMP3 were not measured by immunoblotting, which will be validated in following mechanistic studies.

Taken together, our work investigated the role of TRPA1 age-related changes of cardiac structure and function. The findings indicate that knocking out *Trpa1* accelerated myocardial fibrosis, ventricular dilation, and cardiac dysfunction. Future studies will investigate whether TRPA1 can become a therapeutic target for preventing and/or treating cardiac fibrosis and HFpEF.
